# Brain Digital Slide Archive: An Open Source Whole Slide Image Sharing Platform for AD/ADRD Research and Diagnostics

**DOI:** 10.1002/alz70862_109898

**Published:** 2025-12-23

**Authors:** Margaret E Flanagan, David Gutman, Brittany N Dugger, Lee Cooper, Thomas M. Pearce, Gabor G. Kovacs, Walter W. Kukull, John F. Crary, David Manthey, Sarah Biber, C. Dirk Keene, Claudia Kimie Suemoto, Cody Bumgardner, Peter T Nelson

**Affiliations:** ^1^ Glenn Biggs Institute for Alzheimer’s & Neurodegenerative Diseases, UT Health San Antonio, San Antonio, TX USA; ^2^ Emory University, Atlanta, GA USA; ^3^ University of California, Davis, Sacramento, CA USA; ^4^ Northwestern University, Chicago, IL USA; ^5^ University of Pittsburgh Medical Center, Pittsburgh, PA USA; ^6^ Tanz Centre for Research in Neurodegenerative Diseases, University of Toronto, Toronto, ON Canada; ^7^ National Alzheimer's Coordinating Center, University of Washington, Seattle, WA USA; ^8^ Icahn School of Medicine at Mount Sinai, New York, NY USA; ^9^ Kitware, New York, NY USA; ^10^ University of Washington, Seattle, WA USA; ^11^ University of Washington, School of Medicine, Seattle, WA USA; ^12^ University of São Paulo Medical School, São Paulo, São Paulo Brazil; ^13^ University of Kentucky, Lexington, KY USA

## Abstract

**Background:**

Neuropathological evaluation of brain tissue remains the gold standard for diagnosing neurodegenerative diseases. However, limited data sharing and variability in sample preparation pose significant challenges. Advances in digital pathology and machine learning offer opportunities to standardize approaches and improve diagnostic accuracy.

**Method:**

Building on the Digital Slide Archive (DSA), originally designed for cancer imaging, we are developing the NIH U24 funded Brain Digital Slide Archive (BDSA), a federated, open‐source platform tailored to support research and diagnostics in neurodegenerative diseases. The BDSA supports whole slide images (WSIs) for Alzheimer’s disease (AD) and Alzheimer’s disease‐related dementias (ADRDs), adhering to FAIR (findable, accessible, interoperable, reusable) data principles. Metadata associated with WSIs aligns with the National Alzheimer’s Coordinating Center (NACC) Data Front Door for enhanced accessibility. Workflows to enable standardized sharing of WSIs, annotations, and metadata, while supporting the development and validation of machine learning algorithms across diverse datasets.

**Result:**

Initial development includes integrating WSIs from nine AD/ADRD‐focused research centers, harmonizing data across institutions, and implementing universal data‐sharing agreements. An anonymization tool has been incorporated to scrub labels from WSIs, ensuring donor confidentiality. Required data use agreements and material transfer agreements have been completed for all contributing U.S.‐based sites. Administrative best practices, including regular software testing and standardized protocols, are being applied to create a robust and secure platform. The BDSA will provide a unified resource for research, diagnostics, and training, facilitating scalable and reproducible analyses while preserving institutional data control.

**Conclusion:**

The BDSA aims to democratize access to neuropathological data through a standardized, user‐friendly digital repository. By integrating tools for privacy, data sharing, and machine learning, this platform will enhance understanding of neurodegenerative diseases and foster collaboration across institutions. As a federated open‐source resource, the BDSA has the potential to transform digital neuropathology, driving innovation in AD/ADRD research and diagnostics.

